# Editorial: Heavy metal toxicity in plants: Recent insights on physiological and molecular aspects, volume II

**DOI:** 10.3389/fpls.2022.1016257

**Published:** 2022-10-21

**Authors:** Basharat Ali, Rafaqat A. Gill

**Affiliations:** ^1^ Department of Agricultural Engineering, Khwaja Fareed University of Engineering and Information Technology (KFUEIT), Rahim Yar Khan, Pakistan; ^2^ Key Laboratory of Biology and Genetic Improvement of Oil Crops, The Ministry of Agriculture and Rural Affairs, Oil Crops Research Institute of Chinese Academy of Agricultural Sciences, Wuhan, China

**Keywords:** heavy metals stress, plant genome, phytoremediation, nutrient deprivation, management, cell morphology

Metals with relatively high densities, atomic numbers, or atomic weights are regarded as heavy metals. Among them, some metals are considered essential nutrients (Fe, Co, Zn), or relatively harmless (Ru, Hg, In). However, they can be toxic in higher amounts. At higher levels, heavy metals have abilities to create severe toxic symptoms in crop plants, and therefore, their utilization and uptake are greatly controlled by the plant cells. It has been reported that both essential and non-essential metals produce common toxic symptoms on crop plants, such as chlorosis, low biomass accumulation, photosynthesis, inhibition of growth, and nutrient assimilation that ultimately cause plant death. To combat metal stress, plants have evolved different defense modifications viz., less uptake of metals, phytochelatins binding, and the activation of numerous antioxidants. The first strategy to combat heavy metals in plants is to activate their enzymatic (POD, SOD, APX, CAT, GR) and non-enzymatic (ASA, GSH) antioxidants. The second strategy is to activate their root exudates and cell wall. The third strategy is to utilize plants (phytoremediation) which can absorb the metal content from the soil at higher rates. Lastly, exogenous applications of growth regulators, nutrients, and organic amendments such as ALA, GABA, MeJA, AsA, NPs, MLE, and Se provide some kind of relief to crop plants to combat the hazardous effects of HMs. In a summary, all the amendments under metal/metalloid stress can help the plants to strengthen their defense mechanism and improve their growth in a metal toxic environment. Our Research Topic mainly focuses on plant abnormalities, like alteration in PG&D, nutrient unbalances, and enzyme dysfunctioning due the metal/metalloid toxicity. This Research Topic proved successful in collecting twelve research articles and two review articles based on the impacts of diverse HMs (Cd, Cr, Al, As, Hg) on the plants and alleviation with different organic and chemical amendments.

## Cd-toxicity in plants: Its impact and alleviation

In plants, metal toxicity reduces the uptake and translocation of nutrients and water, and enhances oxidative damage, thus inhibiting plant growth. However, alleviation of different metal toxicity through different plant growth regulators, nutrient application, and organic amendments is an emerging technique. Thus, Yang et al. delineate the beneficial role of 5-aminolevulinic acid (5-ALA) to reduce Cd-toxicity in *Brassica pekinensis* L. In their hydroponic experiment, they prove that emerging plant growth regulator 5-ALA lowers the Cd stress by maintaining redox homeostasis, strengthening the photosynthetic machinery, and improving the expression of different genes related to Cd transport in *B. pekinensis* seedlings. Several studies have shown that the application of methyl Jasmonate (MeJA) induces a stimulatory effect on photosynthetic machinery and thus enhances plant morphology and growth. Manzoor et al. find that the application of MeJA improved the plant growth parameters in different pea varieties under Cd toxicity conditions, such as greater fresh and dry biomass of shoots and roots. Further, enhancement in root biomass was more significant after the application of MeJA as compared to other plant parts. Therefore, the stress-alleviating role of MeJA is attributed to its role in photosynthesis and redox balance of pea plants under Cd stress conditions. Higher Cd absorption has been found higher in wheat (*Triticum aestivum* L.) compared to other cereal crops which can cause high daily Cd intake, thus huge impact on public health. In this context, a pot experiment by Farhat et al. is carried out on wheat seedlings exposed to Cd-stress to explore physio-morphological changes by two bio-stimulants, i.e., ascorbic acid and moringa leaf extract. The results show that Cd-deposition predominantly occurs in roots compared with shoots of wheat seedlings, which is reduced after the application of AsA and MLE. In conclusion, MLE proved to be more efficient to alleviate Cd-induced toxicity compared with AsA treatment.

Further, Cd has been found to interact with plants at physio-biochemical levels, thus resulting in reduced plant growth. Accordingly, Ramzan et al. conduct research to explore the potential of *Moringa oleifera* leaf extract and zinc oxide nanoparticles in alleviating Cd-toxicity in linseed plants. They find that the application of *MLE* and ZnO NPs reduces the negative effects of Cd stress in linseed seedlings by enhancing the contents of antioxidant enzymes. Further, it has been found that amendments like biochar and thiourea have the ability to reduce Cd stress and uptake in crop plants. Therefore, Haider et al. have designed a research trial to evaluate the impact of 1) biochar made of three maize stalks, and 2) three thiourea foliar application rates in alleviating the negative impacts of Cd on the plant growth in maize seedlings. Their study concludes that the application of biochar and thiourea significantly enhanced maize plant growth under Cd-stressed conditions by decreasing Cd concentration in different plant tissues.

## Cr-toxicity in plants: Antioxidants and photosynthetic responses

Chromium (Cr) toxicity in plants is a diverse phenomenon that decreases seed germination, reduces plant growth, inhibits enzymatic activities, and impairs photosynthetic machinery and oxidative imbalances. By keeping in mind the possible beneficial role of different bacteria and nanoparticles in reducing Cr stress in plants, Ma et al. have designed a pot trial to explore the beneficial role of cerium dioxide (CeO_2_) nanoparticles and a bacteria named *Staphylococcus aureus* in alleviating the negative effects of Cr-toxicity in sunflower seedlings. The outcome of this study shows that CeO_2_ nanoparticles and *S. aureus* application significantly ameliorated Cr-induced negative impacts in sunflower plants. In another study, Shah et al. attempt to explain the scarce information on the role of harzianopyridone (HZRP) in the alleviation of Cr stress in *Vigna radiata*. In this study, they primed HZRP at 1 and 2 ppm and find that the application of HZRP under Cr stress enhanced intercellular CO_2_ concentration and photosynthetic rate with enhanced levels of antioxidant enzyme activities and reduced levels of chlorophyllase (Chlase) enzyme in *V. radiate*.

## As and Hg-toxicity in plants: Impact on antioxidants, chlorophyll, and accumulation

Among all the pollutants, Hg and As are known to be among the top five key dangerous metals/metalloids (Hg, As, Cu, Pb, Cd). Though many amendments have been suggested for coping with metalloid toxicity, they are not practically suitable in crop production due to time taking, high cost, or operational complexity. However, the utilization of nanoparticles to potentially reduce metal toxicity in plants has increased in recent times. Therefore, an *in vitro* trial is conducted by Emamverdian et al. to test different levels of ZnO-NPs both alone and in combination with As and Hg in *Pleioblastus pygmaeus*. They find that the application of ZnO-NPs increased antioxidant activities, glycine betaine content, chlorophyll attributes, and eventually plant growth under As and Hg stress in *Pleioblastus pygmaeus.* Further, Nazir et al. have designed a study to investigate the positive role of CaO NPs in reducing As toxicity in two genotypes of barley differing in As tolerance. They state that CaO NPs application under As toxicity significantly enhanced Ca uptake, lowered ROS contents, and reduced As uptake and transportation.

## Al and Pb-toxicity in plants: Impact on plant growth, ROS and transcriptome sequencing

Heavy metal toxicities, i.e., Pb and Al, have been found to cause inhibition in ATP production and enhance ROS and DNA damage. In addition, both metals significantly inhibit germination, root elongation, plant growth, and chlorophyll production, and disturbs transcriptome sequencing. In this context, Ashraf et al. conduct a research trial to evaluate the positive role of exogenously applied gamma-aminobutyric acid (GABA) in modulating the growth and physio-biochemical mechanisms in two genotypes of aromatic rice under Pb stress conditions. They suggest that the application of GABA in rice decreased Pb contents in both shoots and grains, and lowered ROS by enhancing antioxidant enzyme activities compared with Pb-exposed plants. Further, they also find that cultivar GXZ exhibited better performance than NX-18 under Pb-stressed conditions. In recent times, ultrasonic treatment has become an effective strategy to cope with stress under heavy metal toxicity. The study by Bao et al. consists of two treatments, control and ultrasonic seed treatment, in peanut plants. Both treatments were applied with AlCl_3_ in hydroponic conditions. The results depict that plant growth increased significantly with ultrasonic treatment under Al stress. Moreover, a transcriptome study explains that plant signal hormone transduction and transcription factors are enriched significantly in the differentially expressed genes in ultrasonic treatment under Al-stress conditions.

## Se supplementation to enhance crop abiotic stress tolerance

Chemical-assisted approaches, i.e. the application of different essential micronutrients like selenium (Se), is an emerging strategy to mitigate the adverse effects of heavy metals because of their biochemical nature. Hereby, Hasanuzzaman et al. present a review that describes the beneficial role of Se in improving plant stress tolerance under metals stress. They report the improvement in plant growth, and various physiological and biochemical attributes with the application of Se under metal stress. Further, they also suggest that photosynthetic attributes are enhanced by the supplementation of Se under metal stress.

## Phytoremediation mechanisms of potentially toxic elements

The deposition of different toxic elements (Hg, Cd, Se, Cu, As, Pb, Cr) in nature draws significant negative impacts on plants and, ultimately, on humans. Different anthropogenic activities, e.g., gas industries, coal-burning power plants, and agricultural activities, are specific contributors that increase the amounts of toxic pollutants in the atmosphere. The review article by Alsafran et al. reviews different proteomic and molecular approaches to discover the mechanisms underlying plant stress tolerance and bioaccumulation of different pollutants within the plants. According to them, several researchers have elucidated the plants with high-stress tolerance, metal uptake, and metal deposition in different plant parts.

## Response of ZnO NPS on developmental stages of *Phaseolus vulgaris*

Nowadays, extensive use of foliar-applied ZnO NPs has been found to down-regulate different plant growth mechanisms involving photosynthesis, chlorophyll synthesis, and nutrient uptake in crop plants. Similarly, Salehi et al. foliarly applied the ZnO NPs on bean (*Phaseolus vulgaris* L.) plants and find negative effects from ZnO NPs on the differential mechanism involved in the reproductive stage of the plants compared with the salt form.

## Author contributions

BA and RG wrote the first draft of the article. Both authors contributed to the article and approved the submitted version.

## Acknowledgments

We thank all the contributors to this Research Topic.

## Conflict of interest

The authors declare that the research was conducted in the absence of any commercial or financial relationships that could be construed as a potential conflict of interest.

## Publisher’s note

All claims expressed in this article are solely those of the authors and do not necessarily represent those of their affiliated organizations, or those of the publisher, the editors and the reviewers. Any product that may be evaluated in this article, or claim that may be made by its manufacturer, is not guaranteed or endorsed by the publisher.

